# Patients’ experiences on accessing health care services for management of hypertension in rural Bangladesh, Pakistan and Sri Lanka: A qualitative study

**DOI:** 10.1371/journal.pone.0211100

**Published:** 2019-01-25

**Authors:** Helena Legido-Quigley, Aliya Naheed, H. Asita de Silva, Imtiaz Jehan, Victoria Haldane, Benjamin Cobb, Saeideh Tavajoh, Nantu Chakma, Anuradhani Kasturiratne, Sahar Siddiqui, Tazeen H. Jafar, for COBRA-BPS Study group

**Affiliations:** 1 Saw Swee Hock School of Public Health, National University of Singapore, Singapore; 2 International Centre for Diarrhoeal Disease Research, Dhaka, Bangladesh; 3 Clinical Trials Unit, Department of Pharmacology, Faculty of Medicine, University of Kelaniya, Kelaniya, Sri Lanka; 4 Department of Community Health Sciences, Aga Khan University, Karachi, Pakistan; 5 Duke Global Health Institute, Duke University, Durham, North Carolina, United States of America; 6 Program in Health Services & Systems Research, Duke-NUS Medical School, Singapore, Singapore; University of Ghana Business School, GHANA

## Abstract

Hypertension is the leading risk factor for cardiovascular disease and leading cause of premature death globally. In 2008, approximately 40% of adults were diagnosed with hypertension, with more than 1.5 billion people estimated to be affected globally by 2025. Hypertension disproportionally affects low- and middle-income countries, where the prevalence is higher and where the health systems are more fragile. This qualitative study explored patients’ experiences on the management and control of hypertension in rural Bangladesh, Sri Lanka and Pakistan. We conducted sixty semi-structured interviews, with 20 participants in each country. Hypertensive individuals were recruited based on age, gender and hypertensive status. Overall, patients’ reported symptoms across the three countries were quite similar, although perceptions of hypertension were mixed. The majority of patients reported low knowledge on how to prevent or treat hypertension. The main barriers to accessing health services, as reported by participants, were inadequate services and poor quality of existing facilities, shortage of medicine supplies, busyness of doctors due to high patient load, long travel distance to facilities, and long waiting times once facilities were reached. Patients also mentioned that cost was a barrier to accessing services and adhering to medication. Many patients, when asked for areas of improvement, reported on the importance of the provider-patient relationship and mentioned valuing doctors who spent time with them, provided advice, and could be trusted. However, most patients reported that, especially at primary health care level and in government hospitals, the experience with their doctor did not meet their expectations. Patients in the three countries reported desire for good quality local medical services, the need for access to doctors, medicine and diagnostics and decreased cost for medication and medical services. Patients also described welcoming health care outreach activities near their homes. Areas of improvement could focus on reorienting community health workers’ activities; involving family members in comprehensive counseling for medication adherence; providing appropriate training for health care staff to deliver effective information and services for controlling hypertension to patients; enhancing primary health care and specialist services; improving supplies of hypertensive medication in public facilities; taking into account patients’ cultural and social background when providing services; and facilitating access and treatment to those who are most vulnerable.

## Introduction

Hypertension is the leading risk factor for cardiovascular disease and leading cause of premature death globally [1]. In 2008, approximately 40% of adults were diagnosed with hypertension, with more than 1.5 billion people estimated to be affected globally by 2025 [2]. The problem is particularly serious in South Asia, with trends data indicating an increase in age-standardized levels of blood pressure (BP) [3]. The rising prevalence of hypertension is attributed to ageing, population growth and behavioral risk factors such as unhealthy diet, excessive alcohol consumption, reduced physical activity and exposure to prolonged stress [3]. However, whilst hypertension is largely a preventable condition and many effective treatments are available [1], the majority of individuals in low and middle income countries remain undiagnosed, untreated and/or uncontrolled [4, 5].

Previous systematic reviews have identified multiple challenges to effective hypertension prevention, management, and control [6, 7]. Barriers at the health system level include poor or absent screening programs, inaccessible health care facilities, high costs of treatment, poor provider-patient communications, and patients’ distrust in the services [7]. Notwithstanding the relevance of these results, the reviews highlight the need for more qualitative research in low- and middle-income countries (LMICs), since the majority of studies identified were from high-income countries, and from those that were conducted in LMICs, only a handful adopted a qualitative methodology [6, 7]. Furthermore, there is a pressing need to understand key barriers to hypertension management in LMICs since the prevalence is higher, they encompass a large population, and health systems are more fragile [5].

This qualitative study aims to address a gap in the literature by identifying health system and population barriers to accessing hypertension care services in rural communities in Bangladesh, Pakistan and Sri Lanka. This study is part of an ongoing cluster randomized controlled trial study evaluating a multicomponent intervention (MCI) specifically comprised of 1) home health education (HHE) by government community health workers (CHWs): plus 2) BP monitoring and stepped-up referral to a trained provider using a checklist; plus 3) training public and private providers in management of hypertension and using a checklist; plus 4) designated hypertension care coordinators in government clinics, plus 5) performance-based compensation to lower systolic BP among adults with hypertension versus usual care.[8]. A key qualitative objective of the study is to identify patients’ experiences of symptoms, awareness of prevention, as well as knowledge and control of hypertension, especially as it relates to adherence to lifestyle and medication. This paper reports the qualitative analysis of the above-mentioned objectives, including barriers to accessing services from the perspective of hypertensive individuals.

## Materials and methods

### The case study settings: Bangladesh, Pakistan and Sri Lanka

#### Bangladesh

Overall prevalence of hypertension in Bangladesh is estimated to be 26.4%, with higher prevalence in women (32.4%) than men (20.3%) [9]. Studies have reported less than one-third of patients with hypertension having controlled blood pressure [10, 11]. The population of Bangladesh is 162.9 million [12], of which about 66% is rural. The Bangladeshi health system is highly centralized with four main actors delivering and funding healthcare: government or public sector, private sector, nongovernmental organizations (NGOs) and donor agencies [13]. The public sector is responsible for policy and for the provision of health services offered through community clinics and hospitals. Bangladesh has a far-reaching primary health care infrastructure, with CHWs providing immunization and basic preventive child care services, and clinics traditionally geared towards infectious diseases. However, the CHWs do not provide screening or education on lifestyle approaches for prevention of hypertension. Recently the government created non-communicable disease (NCD) corners in health facilities, however these facilities lack equipment and trained staff to screen patients for NCDs. In 2011, total health expenditure as percentage of the gross domestic product (GDP) was 2.8% [14].

#### Pakistan

The National Health Survey of Pakistan 1990–1994 estimated that 18% of the adult population and 33% of people over the age of 45 years were hypertensive, the vast majority (70%) were unaware and control rates were less than 10% [15]. Subsequent studies in 2003–2004 in urban Pakistan indicated hypertension prevalence of 40% among individuals aged 40 years or older [16]. Pakistan is the 6^th^ most populous country with a population of 193 million, 61% of which is rural [12]. Pakistan’s health system includes both public and private for profit and non-for profit actors. The private sector plays a dominant role especially in urban areas. The public health care infrastructure includes community services, rural health centres, tehsil hospitals and district and tertiary hospitals. However, there are marked urban-rural disparities in both healthcare delivery and human resources. Nearly 80% of the population pay out of pocket expenditures to access services, which poses a significant problem for chronic lifelong therapies including anti-hypertensive medication use. Despite the establishment of the government-funded Lady Health Workers’ Programme (LHWP) in 1990, and currently with more than 100,000 LHWs to provide immunization and family planning services, as in Bangladesh, this cadre of CHW does not have the mandate (or training) to provide NCD or hypertension screening or related education. Overall, health expenditure only represents 2.6% of GDP- the lowest in South Asia [12].

#### Sri Lanka

A 2014 study estimates that the prevalence of hypertension in all adults in Sri Lanka is 23.7% [17]. Another study of 4,400 adults between 35 and 64 years of age, reported that of the known hypertensives, 19.5% were not on anti-hypertensive medication and only 32.1% were controlled [18]. The population of Sri Lanka is 21.2 million, of which 81.5 is rural [19]. Sri Lanka has a universal health care system with a comprehensive public network providing preventive, treatment and management services at a relatively low cost. The government sector provides healthcare and medications free to all citizens covering in-patient treatment as well as preventive care [20]. However, the public sector faces challenges such as, inadequate human resources and substantial differences in standards between rural and urban areas. Public Health Midwives (PHMs) play a crucial role in delivering services at community and primary healthcare level. These health workers have mainly focused on midwifery, although recently their role has been expanded to include providing advice on preventive measures. In 2012, Sri Lanka’s expenditure on health was around 3.2% of GDP [19].

### Conceptual framework

The approach to conceptualising access to care from the patient’s perspective in this paper is underpinned by Levesque et al’s framework [21] which positions access at the interface of health systems and populations. Access is conceived in a much broader sense, as it not only relates to accessing services, but includes the pathways the patient traverses from identifying health needs, to having their needs for accessing services fulfilled. Along this pathway, it is important to understand how patients seek services, how they reach them, and how these are obtained and used. This conceptual framework identifies factors that may impact access and focuses on both those related to the health system (e.g. institutions, organizations, providers) and those present at population level (e.g. individual, household, community). The authors conceptualize access under five key dimensions, namely: 1) Approachability; 2) Acceptability; 3) Availability and accommodation; 4) Affordability; 5) Appropriateness. The novelty of this framework is that Levesque et al identify these dimensions with corresponding abilities from the perspective of the patient. The corresponding abilities include: 1) Ability to perceive; 2) Ability to seek; 3) Ability to reach; 4) Ability to pay; and 5) Ability to engage. The key in operationalizing the framework is to understand that these set of dimensions interact with one another and both accounts need to be considered in order to plan appropriate integrated services. For example, ‘Availability’ relates to the existence of health services that can be reached and are organized with adequate human resources. From an individual perspective, ‘ability to reach’ refers to patients having the means to access those facilities and to do so in a timely manner. This framework has been previously adopted in a variety of settings (e.g. Brazil, China, South Africa) to conceptualise access to health care describing the broad determinants that encapsulate demand and supply-side factors whilst facilitating the operationalization of access to health care including both experiences, that of obtaining care but also that of benefiting from the services provided [21] [22] [23] [24].

### Study design and sampling

A total of 60 semi-structured interviews were conducted in rural areas in Bangladesh, Sri Lanka and Pakistan. Interviewees were selected purposively from the COBRA-BPS trial study and included selecting interviewees based on their age, gender, hypertensive status (controlled, uncontrolled), socioeconomic status, and distance of household from government clinic.

The study was conducted in Bangladesh, District Tangail and District Munshiganj; Pakistan, District Thatta, and Sri Lanka, Puttalam District. The unit of randomization was a cluster defined by 250–300 households as defined by local administration. Clusters were selected randomly for inclusion in the study, and randomized to intervention and usual care stratified by distance (a distance of 2 km or less will be defined as ‘near’, and more than 2 km as ‘far’) from the government clinic.

Hypertensive individuals were recruited from 15 clusters (5 in each country) randomized to intervention based on age, gender, and hypertensive status (controlled, uncontrolled), and near/far clusters. Criteria for hypertension was either persistently elevated BP (systolic BP ≥140 mm Hg or diastolic BP ≥90 mm Hg) from each set of last 2 of 3 readings from 2 separate days; and/or currently maintained on anti-hypertensive medications. Interviews were conducted between April 2016 and March 2017.

All locations included in the study were rural areas. In Bangladesh, the study involved interviews with 20 patients in Lauhajang, Sirajdikhan, Tangibari sub-district in Munshigonj district and Mirzapur in Tangail district. Interviews were conducted in Bangla and then translated in English. In Pakistan, the 20 interview participants selected lived in either Thatta, Gujjo, or Jungshahi, Sindh, all of which are located in District Thatta. Interviews were predominantly conducted in Sindhi. In Sri Lanka, 20 interviews were conducted in five rural areas (Anamaduwa, Chilaw, Mahawewa, Mundel and Pallam). The site PIs and project coordinators with experience in qualitative research were trained in the common protocol and interview guide by a qualitative expert (HLQ) until all teams were comfortable with the purpose and the interview guide. Prior to interviews beginning, the questions in the interview guide were translated into local languages and pre-tested. Audio recordings and notes were taken during each interview and reviewed by the site PI. The in-depth interviews were recorded and subsequently transcribed into local dialect. The transcripts were then translated into English. All translations were reviewed line by line by a bilingual interviewers in each country and double-checked by the site principal investigators (AN, AdeS, I). All interviewers were bilingual and trained in conducting the interviews using probes developed by HLQ, AN, AdeS, IJ, and THJ in study-group meetings facilitated by HLQ, and detailed sessions at the icddr, Bangladesh, University of Kelaniya, and Aga Khan University, and investigators’ meeting at Duke-NUS Medical School Singapore. The training included a description of the research protocol, qualitative study method, and principles of ethical research.

Interviews were conducted in the respondent’s home. Characteristics of respondents are detailed in Table 1 and S1 Table includes the Interview Topic Guide.

**Table 1:** Patients characteristics by country.

Patient Characteristics	Female	Male	Total
**Bangladesh**			
**Age Range**			
**40–50**	4	0	4
**51–60**	3	8	11
**61–70**	2	2	4
**71+**	0	1	1
**Location**			
**Lauhajang**	2	3	5
**Mirzapur**	3	2	5
**Sirajdikhan**	1	4	5
**Tangibari**	3	2	5
**Management of Hypertension**			
**Controlled**	5	3	8
**Uncontrolled**	4	8	12
**Sri Lanka**			
**Age Range**			
**40–50**	1	2	3
**51–60**	1	2	5
**>60**	8	4	12
**Location**			
**Anamaduwa**	3	1	4
**Chilaw**	3	1	4
**Mahawewa**	2	2	4
**Mundel**	3	1	4
**Pallam**	2	2	4
**Management of Hypertension**			
**Controlled**	5	1	6
**Uncontrolled**	6	4	10
**Not clear**	2	2	4
**Pakistan**			
**Age Range**			
**40–50**	3	4	7
**51–60**	3	1	4
**61–70**	3	4	7
**70+**	2	0	2
**Location**			
**Thatta**	5	7	12
**Gujjo**	1	1	2
**Jungshahi**	5	1	6
**Management of Hypertension**			
**Controlled**	10	8	18
**Uncontrolled**	1	1	2

All respondents were given an information sheet and were asked to sign and date a consent form. Consent was also obtained for audio-recording. All interview materials were stored ssecurely to assure confidentiality. Confidentiality was ensured by giving each participant the option of not being quoted, even anonymously, in the study and subsequent publications. Efforts were made to conduct the interviews in a private space that was deemed suitable for the respondent.

#### Analysis

This study is firmly grounded in an interpretive approach, where the knowledge produced accounts for patients’ perspectives, the meanings associated to their social world, and how they construct their reality around accessing health services.

Four research team members (VH, BC, ST, HL-Q) coded interviews using both an inductive and deductive approach and thematic analysis. THJ, and team members in each country (AN, IJ, SS, AdeS, AK) agreed on the codes, themes and subthemes. Once the key themes were identified, these were compared across countries aiming to identify patterns in the data, but also allowing for differences in each country to emerge and be considered in the analysis. Analysis was carried out adopting techniques from grounded theory, such as the use of the constant comparative method, conducting line-by-line analysis of each interview and identifying deviant cases. Data from the interviews was assessed for quality at each level of analysis first by the interviewer, and then the site PI. THJ performed random checks of transcripts. Data quality was assessed by reviewing the variety of responses, elaboration of answers, and repetition of opinions, and discussed in meetings with site teams. Undetected or inaccurate interview analysis was discussed among HLQ, THJ, site PI and the coder to avoid mis- interpretation of the interview results, and any inconsistencies were resolved by repeat visit to the participant for clarification, if needed.

QSR NVivo 11 Software was used to analyze the data. Thematic saturation was assessed first within each site. We agreed within sites that data should be collected until PIs and data collectors were confident that no new patterns and themes were emerging from the interview data. Discussions among all the researchers were key to ascertain thematic saturation for each of the themes identified in each country. All site PIs suggested that thematic saturation had been reached before reaching interview twenty, but we all agreed to continue interviewing to achieve that target number. All researchers agreed that we were able to provide a thorough analysis of all the themes identified since we had a variety of responses with a wide range of opinions and a diversity of views. We established thematic saturation when we identified ‘rich information’ in each of the themes and no more relevant themes were emerging from the data.

Each quote contains the interview number and code letter (F for Female, M for male) and the country of origin for each participant (B for Bangladesh, PK for Pakistan, SL for Sri Lanka). In this paper, all names are pseudonyms and identifying data have been removed to maintain confidentiality.

## Results

**Fig 1:**
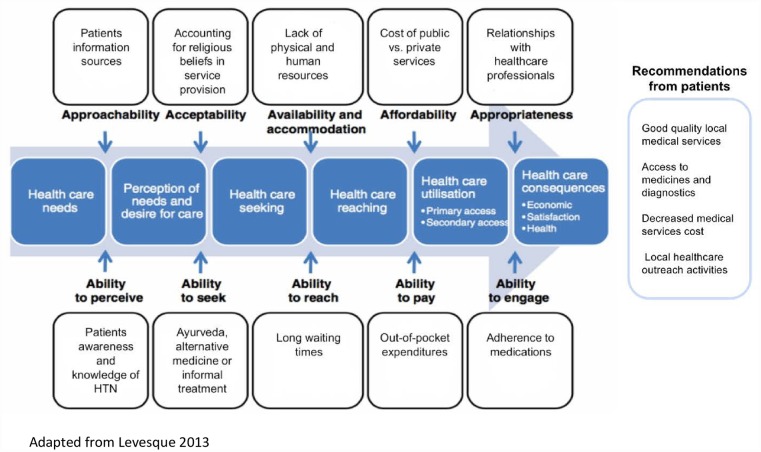
Conceptualization of Levesque et al. through patient perspectives from Bangladesh, Pakistan and Sri Lanka.

We compared the data and main themes identified in Bangladesh, Sri Lanka and Pakistan and organized our results under six key themes as there was good quality data to illustrate the pre-determined theoretical categories. The first five themes follow Levesque’s et al key access dimensions with their corresponding abilities from the perspective of the patient and the sixth theme was developed inductively to report on the needs for improvement as expressed by participants is shown in Fig 1.

Ten subthemes were identified inductively. The first theme ‘Approachability and ability to perceive’ describes in three subthemes patients’ experiences of hypertension symptoms (if any); patients’ awareness and knowledge of hypertension; and sources of information drawn upon to inform decisions. The second theme ‘Acceptability and ability to seek’ discusses the use of alternative medicine and religious beliefs and practices, as both cultural and religious factors influenced how different groups accepted the services provided. The third theme ‘Availability and accommodation and ability to reach’ explores the inadequacy of physical and human resources, long waiting times, and shortages of medications. The fourth theme ‘Affordability and ability to pay’ reports on excessive out-of-pocket expenditure; and the differences in experiences reported when accessing private versus public services. The fifth theme ‘Appropriateness and ability to engage’ reports on how medication taking behavior influenced adherence; and patients’ experiences with healthcare professionals including community health workers, doctors and pharmacists. Finally, the sixth theme summarizes what patients reported could be improved in the provision of hypertension services.

### ‘Approachability and ability to perceive’: Patients’ awareness of hypertension and information sources

‘Approachability and ability to perceive’, in this context, refers to patients’ opportunities to identify hypertension services and the sources of information drawn upon to inform their decisions [21]. Linked to approachability is the concept of ‘awareness’. This related concept alludes to patients’ awareness of hypertension and whether, as a result of this understanding, they perceive that care is needed.

#### Patients’ experiences of symptoms associated with diagnosis of hypertension (if any)

Overall, patients’ reported symptoms (or absence of them) across the three countries were quite similar. Most patients recalled experiencing mild to moderate symptoms when asked whether they felt any warning signs prior to the diagnosis of hypertension. The symptoms reported might be attributable to hypertension, or not, but included “faintish feeling”, “feeling like drunk”, “drowsiness”, “shortness of breath”, “palpitations”, “fatigue and loss of energy” and “headache”. Several participants reported more acute symptoms such as “vomiting”, “extreme weakness”, “fainting”, and “having the body paralyzed”. In a few instances in the three countries, patients reported not having any symptoms prior to being diagnosed.

#### Patients’ awareness and knowledge of hypertension

Most patients reported awareness of hypertension prior to diagnosis, but low knowledge of the specifics on how to prevent it or treat it. Particularly in Pakistan, several respondents reported having little knowledge prior to being diagnosed. The following quote summarizes the sentiments of those patients:

“*Nobody ever educated us about the nature of the disease and the implications if it is not controlled. I have it and am living with it*.”

(*I5PKM*)

None of the interviewees reported being aware that hypertension is normally asymptomatic and there was little awareness of the possible consequences prior to being diagnosed. Those that reported possible consequences described a variety of beliefs s such as heart attack, stroke, headache and neck pain.

Some of the reports of those who knew about the condition were expressed in a dramatic manner and emphasized the fact that hypertension could lead to very serious conditions. This was particularly the case in Bangladesh where several participants used a similar example to report what could happen. It was suggested that untreated hypertension could lead to *‘the neck vein tearing off’ or ‘the neck vein rupturing’*.

Across the three countries there were limited reports on what patients believed to be the causes of hypertension, however some patients in Bangladesh and Pakistan reported that labour and stressful situations were considered important factors.

Several accounts were also provided of stressful events including fights between family members or the death of children as possible triggers to developing hypertension. The following example was provided by Hasan, in Pakistan, who described how the deaths of three of his four children made him very ‘weak’, whilst suggesting that these ‘unprecedented events’ might have contributed to his diagnosis of hypertension:

“*I am Hypertensive for last 12 years. I lost my two sons one at the age of 40 years from electrocution and other died of unknown case even more younger. One of my daughter died of breast cancer and TB. All these unprecedented events made me very weak and I was not feeling good, so my son took me to the doctor and he checked my BP and experienced frequent headaches which made me unable to do house chores*.”

(*I19PKM*).

Overall, when comparing the three countries, patients’ perceptions of hypertension were mixed. Some patients downplayed its significance whilst others recognized it was a serious condition.

#### Sources of information drawn upon to inform decisions

Patients reported several sources of information available to them besides those provided by community health workers, clinicians and pharmacists. For some patients, prior knowledge was obtained through personal experience, from family members and friends, and ‘hearsay’. Patients reported these sources providing information on lifestyle changes, medication adherence and options for access to care.

In all countries, ‘hearsay’ plays an important role in shaping participants perceptions. The next quote from Sri Lanka illustrates an example of such instances:

“*I didn’t know any details regarding this disease and such. If blood glucose is high, we die. If blood pressure is high, we die. Those things I’ve heard. But I didn’t know any details about the disease*.”

(*I01SLF*)

Few participants in the three countries could recall any prevention campaigns to increase knowledge about hypertension. As for information provided by healthcare professionals, most reported either CHWs or pharmacists playing this role. For example, in Pakistan, advice was typically provided by LHWs. There were no reports of outreach activities for hypertension or NCDs in Bangladesh; although there were accounts of government health workers, such as Health Assistants (HAs) and Family Welfare Assistants (FWAs), visiting homes to provide vaccinations and family planning.

However, in Sri Lanka, several sources ‘for educating’ outside of families and communities were mentioned. These ranged from the advice provided by healthcare professionals (including Public Health Midwives), special programmes run in the clinics, to the Ministry of Health, and TV programmes. There were several reports on the need to engage in healthy behaviours besides the need to adhere to the medication.

### ‘Acceptability and ability to seek’: The use of Ajurvedic, alternative medicine and religious beliefs

‘Acceptability and ability to seek’ refers to the cultural and social factors that influence how different social groups accept the services provided to them. These aspects are normally shaped by culture, norms and values in a society [21]. The use of Ayurveda and/or alternative medicine and the need for the health system to take into account participants’ religious beliefs and practices emerged as key themes in the three countries.

#### The use of Ayurveda, alternative medicine, or informal treatment

Some participants in each country reflected on the use of Ayurvedic medicine, alternative medicine, or informal treatment, when exploring adherence to medication. Ayurvedic medicine was more frequently mentioned in Sri Lanka, whilst informal treatment was most often referred to in Bangladesh. Those participants who reported using Ayurveda or alternative medicine revealed taking this medication together with allopathic medication for hypertension. In all cases, access to ‘traditional medicines’ and ‘modern medicine’ was separated, with patients seeking and engaging with different providers, in different settings, for their alternative and allopathic needs respectively. The following is a quote from a participant who reported taking allopathic medicine for hypertension as well as ayurvedic medicine for other conditions.

*R: I took ayurvedic medicine two times for my abdominal pain. I went to Jamurki and Mirzapur but didn’t work. So he (husband) brought ayurvedic medicine. After taking that my abdominal pain was gone*.

*I: Any other medicine for HTN…*.

*R: No. Only took allopathic medicine for hypertension*.

(*I10BDF*).

#### Taking into account religious beliefs when providing services

Several participants, particularly in Pakistan and Bangladesh, mentioned a lack of understanding on how to cope with hypertension during Ramadan, None of the participants were provided with advice on how to manage their condition and how to adapt their diet while fasting. Most participants reported discussing these issues with other patients who had hypertension or acting upon what they had heard from ‘others’ or from TV talk shows:

“*The doctor didn’t (tell me what to eat during Ramadan) but when any talk show runs on TV we watch that and try to follow that*. *(I19BDF)”*.

The next quote illustrates how patients may feel conflicted on what to do during Ramadan. Whilst the doctor has forbidden him to drink milk, he feels he needs to drink it during Ramadan. Overall, what transpires from this account is the lack of information and communication with his doctor:

*I: Did the doctor give you any advice to control hypertension or about diet or physical activity*?

*R: No, he didn’t say anything about that. He didn’t tell anything about diet. We know I should not eat meat or drink milk. These foods increase blood pressure. The doctors say, these are totally forbidden for the hypertensive patients*.

*I: How do you know about it*?

*R: I heard from the other hypertensive patients. They told me not to drink milk but I must have milk in this Ramadan* […]

(*I04BDF*)

### ‘Availability and ability to reach’: Mounting difficulties in accessing services

‘Availability and ability to reach’ relates to the existence of health services directed towards hypertension management. This is linked to the concentration and distribution of services and is of particular importance in a rural setting. From an individual’s perspective, having the means to access facilities and doing so at an affordable cost are key components [21].

#### Inadequate physical and qualified human resources

Most patients in the three countries mentioned several difficulties in accessing services. The main barriers to access reported by participants were the lack of local services, long transportation and distance, the busyness of doctors, and the cost of the services. These challenges were particularly emphasized in Pakistan, as a female participant recounted:

“*There is complete dearth of health care facilities here in our village. We do not have enough qualified health care providers and we can’t afford to rush to bigger cities like Hyderabad and Karachi because of our family commitments as it involves travelling the whole day. The doctor I used to visit can only check my B.P. as no other services are available there*.”

(*I12PkF*)

Similarly, some interviewees in Bangladesh also reported challenges in accessing available government services due to distance. *“I don’t go there [the government hospital] because it is too far*.*”(I03BDM)*

Several patients, again more often in Pakistan, complained about the lack of specialist services. One particular patient felt his chances of survival without a specialist doctor in the area were low during his myocardial infarction and mentioned: *“At least provide us with a specialist who can serve us with the immediate life-saving treatment*.*” (IPk06M)*.

#### Long waiting times and shortages of medications

Across the three countries, not only long distances to facilities, but also waiting times once they arrived at government facilities, were described as a normal and expected occurrence. As described by a female participant in Sri Lanka:

*I: At what time do you leave from here in the morning*?

*P: It’s around 7.30–8 a.m. I have to wait there until 1 or 2 p.m. on some days*.

(*I14SLF*)

Other constraints faced in government services included shortage of medications. The next quote from Bangladesh illustrates the many sentiments reported in the three countries:

“*The government doctors neglect the patient…they do not give any medicine. We need to go there and stand, buy tickets and many other hassles. That’s why I do not go there*.”

(*I13BDF*)

Finally, respondents iterated that time consumed from travelling all day kept patients from caring for children, working and earning a wage, and providing for their family. Several patients reported they stopped seeing their doctor due to the travel and time commitment:

“*Travelling from remote areas to the city is always a very daunting task and I can’t afford the travelling expenses along with the medications which they prescribe, so I decided not to visit*.”

(*IPk02M*)

### ‘Affordability and ability to pay’: Excessive out-of-pocket expenditure and private vs. public services

‘Affordability’ refers to the economic capacity of people to spend time and resources to access health services. Significant components include the ‘ability to pay’, which entails the possibility of affording the services needed without exposing people to financial hardship and being able to endure the opportunity costs involved in loss of income [21].

#### Out-of-pocket expenditure

Most patients in the three countries reported on the high cost of care, as well as the cost of medicines, although to varying degrees. The vast majority of respondents reported having no form of health insurance, particularly in Pakistan, which forced out-of-pocket payments for all services and medication. The following example illustrates how the high cost of medication was a common occurrence:

“*We have to pay out of our pocket for every service that we received there and even now on my monthly medications, which costs around Rs.2000/month (19 USD). It is very difficult for us to make our both ends meet with this health problem*.”

(*I06PKM*)

The previous quote illustrates how many participants were finding it difficult to ‘make ends meet’. For most participants, it is not only paying for the medication, but the fees for services and tests, as well as the cost for the ambulance, among other additional costs. As reported by Hasan:

*“Commuting from Thatta to Hyderabad not only financially burdened us in terms of arranging an ambulance*, *purchasing medications and undergoing investigations*, [25] *paying for the doctor’s fee added fuel to the fire”*

(*IPKM*).

Patients also reported on the economic opportunity and indirect costs of treatment, especially in accessing specialized care in urban areas. As described by a participant in Sri Lanka:

“*By bus or vehicle. If going by bus have to start at 4.30am and then reach there by 6.30 and after the consultation come home by 10am. If going by vehicle, it costs around Rs.5000 for Fuel (32 USD). Usually I try to go by bus as much as possible*.”

(*I02SL*)

Patients also mentioned that cost was a barrier to disclosure of illness, as well as adherence to medication. The next quote is an example of the several instances where patients reported stopping their treatment or not obtaining a refill of their prescription due to cost: *“I have stopped taking [medication] due to economic problems*. *I don’t always have the money (I17BF)*.*”*

Most participants reported family members helping each other in time of financial hardship. It was quite common for participants to mention that their next of kin, particularly adult children, contributed to their health care expenditure.

Several patients also reported on medication not being prohibitively expensive. These accounts mainly referred to those instances when the government provided the medication for free, *“I haven’t spent too much for HT treatment*. *I didn’t buy any medicine; it was government medicine (I07BM;”* or when it was bought at the pharmacy, *“I didn’t spend that much of money*. *One strip of medicine costs 8 taka (0*.*1 USD)*. *I have no other expenses (I02BF)*.*”*

#### Accessing services: Public versus private

Participants reported overcoming barriers to medication shortages or long waiting times in the public sector by accessing private providers or local pharmacies. The next quote is an example of the many reports in the three countries:

“*If I go to the private place then there is no issue at all. Only four or five are there. We can go inside in no time and can come back taking treatments. But the medications are expensive*.”

(*I17SLF*)

As suggested in the previous quote, whilst accessing the private sector might be an alternative, the costs involved ‘are expensive’. Most participants reported accessing the private sector for medication or small procedures. In some cases, participants mentioned refusing follow up in private facilities due to cost, or even reported only disclosing some health problems, as they could not afford all the recommended medications. The following quote is an example of such accounts:

“*The cost is very high…suppose if I go to the [private] doctor and tell everything about all of my problems then the cost is like 800 or 1000 taka (9.7 or 12.2 USD). So I do not tell about all of my problems. I just tell about the high blood pressure and take medicine. I just tell about the major problems and then I take medicine for that…if you tell more problems then the doctor will prescribe lots of medicines*.”

(*I04BDF*)

### ‘Appropriateness and ability to engage’: Adherence to medication and relationship between providers and patients

‘Appropriateness’ relates to the experience of care once patients access services. This includes the quality of the services provided and the nature of the interpersonal relationships between providers and patients. At a patient level ‘ability to engage’ refers to whether the person is fully engaged in care and interacts with the services provided [21]. When looking specifically at hypertension one of the key components is whether the patient adheres to medication and physical activity recommended.

#### Medication taking behavior that influences adherence

Overall, in the three countries, most participants reported no problems in taking their hypertension medication. The majority of patients who were diagnosed reported being prescribed medication and educated on lifestyle changes to mitigate hypertension, such as dietary modifications. Once they had visited a clinician, patients mentioned knowing of the need to take medication and linked that directly to feeling better. Patients also reported knowing of multiple medications available for hypertension treatment.

However, there were also many reports in the three countries of patients’ not adhering to the prescribed medication. The following quote from Pakistan is an example of the many reports: *“I usually do not take the medications regularly (I20PKM)”*.

A majority of patients reported they became non-adherent to prescribed medications after they felt better and only take medication when they become symptomatic. Some explained that taking too much medicine for hypertension is not good or reported simply forgetting to take their medication on a daily basis. The next quote illustrates how in some cases a combination of reasons leads to non-adherence:

“*As I feel well now so I don’t take medicine. I can understand when my blood pressure becomes high. But I do not take always blood pressure medicine because taking too much medicine for hypertension is not good. I am not taking medicine because I am feeling well for the last 6 months*.”

(*I05BDM*)

A few patients reported on facilitators for adherence, primarily the role of family members. Among those, there were many reports of patients being reminded to take their medication by their children and spouses.

#### Patients’ experiences with healthcare professionals

Many patients reported on the importance of the doctor-patient relationship and mentioned valuing doctors who spent time with them. However, some patients reported that, especially in government hospitals, doctors did not have enough time to spend with them. As Nusrat reported:

“*I needed more advices…such as we went to the MBBS doctor at the government hospital. He just listened to me and prescribed medicine but didn’t ask anything about my problems. We hoped to get more information but they weren’t encouraging*.”

(*I19BDF*)

Patients reported in all countries their unmet need for information and being unable to communicate their concerns, as well as a lack of continuity of care and follow up. As a patient in Bangladesh emphasized:

“*I didn’t go to the government hospital because they don’t care about patients. They don’t give enough time there. Just provide a prescription and ask to take medicine accordingly. I went to the government hospital few times and tried to talk with the doctor but he stopped me. Then he just gave me a prescription. I told him that I had more problems but he didn’t listen that. That’s why I don’t prefer to go there*.”

(*I18BDM*)

There were also some reports of trusting relationships and good experiences. This was the case when the doctor showed a more familiar and caring attitude, when patients were examined well, and when doctors spent sufficient time to talk with the patient. The following quote exemplifies when positive reports were mentioned:

*I: Does he talk to you well*.

*P: He talks to me very well. Talks well and knows. He knows daughter as well*.

*I: So … do you have a faith on that doctor*.

*P: Yes, I have a very good faith on that doctor*.

*I: Now when you go to take medicines, how long does he spend with you to talk*?

*P: As I think, with me … a considerable time … about 20 minutes he talks with me*.

*I: Does he examine you well*?

*P: Yes…examines we very well*. *Examines spine, legs and everything and … We go there with reports. He asks a lot of information as you all do, he asks a lot of questions*

(*I12SLF*).

Amongst patients who reported preferring private care to public, the majority reported time spent with the doctor as factor in their assessment that private care is of higher quality. Patients reported on the quality of private health care as a motivating factor to access:

“*I seldom go to government hospitals. I go to private doctors mostly…because we don’t need to stand in line there, or buy tickets. We don’t need to do many things there like no medicine so buy medicine, don’t need to go outside to buy injection if need. I always go private doctors. I seldom consult government doctors. The treatment is better at private hospitals*.”

(*I13BDF*)

### Recommendations and areas for improvement as reported by participants

Patients identified multiple unmet needs and recommendations for better provision of hypertension treatment and management. Patients in the three countries reported the need for accessing doctors, medicine and diagnostics, especially in government health care settings, as well as the need for decreased cost for medical services. Respondents offered suggestions such as providing CHWs with functional manometers, increasing specialist doctors locally, and increasing the quality of government-operated clinics. Additionally, patients suggested lowering medication costs. Some respondents suggested that it should be the government’s responsibility to provide medication:

“*The government should take necessary steps to solve this problem. The government has given us a hospital so they need to supply all the medicine*”

(*I18BDM*)

Patients also described welcoming the prospect of greater health outreach into their communities. Others reported that this type of outreach could provide an opportunity to link rural and poor individuals to healthcare, address the unmet need for greater prevention education, lifestyle education and assessments. The next quote illustrates how health outreach activities can be of help, particularly for those most in need:

“*It will be very good…such as there are many people at the rural areas still who don’t dare to go to the doctors*. *Many people can’t go for money*. *Many people don’t know where to go*.”

(*I14BDM*)

The next quote also highlights the willingness to receive more training on hypertension management from the Public Health Midwives in Sri Lanka, but a recognition that this is yet to happen:

*P: If we have more advices that will be good*.

*I: More advices will be good. Right*?

*P: Yes*.

*I: If midwife come and teach*?

*P: That will be good. But we do not get*.

*I: You need more knowledge*?

*P*: *Yes*

(*I10SLF*)

Finally, patients also provided insight into suggestions outside of the health care system. Patients, mostly in Pakistan, often answered with a desire for increased access to clean water, electricity, and employment opportunities.

(See Tables 2 and 3 for a summary of the key findings with themes and subthemes and relevant quotes).

**Table 2:** Key themes with subthemes and relevant quotes.

‘Approachability and Ability to perceive’: Patients’ awareness of hypertension and information sources	
Patients’ experiences of symptoms (if any) and beliefs (Sri Lanka)	Yes, I feel it. I get a headache, faintish feeling. Then when I go to the doctor, he examines and give medicines.
Patients’ awareness and knowledge of hypertension treatment, management and control (Bangladesh)	“If I forget to take medicine then I feel neck pain, headache, discomfort in head, then I try to drink lime sherbet if lime is available at home that time. After that the blood pressure reduces a little then I make my sons buy me medicines.” IDI-BD-04 Age 50-59-F
Sources of Information drawn upon to inform decisions (Pakistan)	“LHWs were and currently are the source of providing awareness and information.” (I1PKM)
‘Acceptability and Ability to Seek’: the use of Ayurveda medicine and religious beliefs	
The Use of Ayurveda, alternative medicine, or informal treatment (Bangladesh)	R: I took ayurvedic medicine two times for my abdominal pain. I went to Jamurki and Mirzapur but didn’t work. So he (husband) brought ayurvedic medicine. After taking that my abdominal pain was gone. I: Any other medicine for HTN…. R: No. Only took allopathic medicine for hypertension. In: Many people take alternative medicine for hypertension…. (IBD10)
Taking into account Religious beliefs when providing services (Bangladesh)	I: Did the doctor tell you about eating during the Ramadan? R: The doctor didn’t but when any talk show runs on TV we watch that and try to follow that (I19B)
‘Availability and Ability to reach’: mounting difficulties in accessing services	
Long waiting times (Bangladesh)	“The doctors there don’t give enough time. We need to wait for long time in queues to buy ticket. We need to wait to meet the doctor as well. We can’t stand for long for illness.” IDI-BD-19
Shortages of medications (Pakistan)	“There is absolutely no health care facility here in our village. Apart from checking the B.P. there is no other thing that the RHC (Thatta) doctor has to offer. No EKG machines for cardiac emergencies. No lifesaving medicines.” (Pk06, Male, 40–50 ys, Controlled HTN)
Lack of physical and human resources (Bangladesh)	“I told him details and he gave me enough time also…there are some doctors who do not give enough time or ask to come later but this doctor is not like that.” IDI-BD-10
‘Affordability and Ability to Pay’: out-of-pocket expenditure and merits of private vs public services	
Out-of-pocket expenditure (Bangladesh)	“Some tests like blood test, urine test should be available in the UHC but here those tests are not available. Suppose it takes one hundred taka for urine test at the outside, so if they took thirty or fifty taka for the same test at the UHC then the public would be benefitted.” IDI-BD-07
Out-of-pocket expenditure (Pakistan)	“Medicines should be provided to us by the government as the doctors prescribe expensive medications which is an extra burden on my son’s family.”(Pk14, Female, 40–50 ys, Controlled HTN)
Accessing services: Public versus Private (Pakistan)	“If I go to the private place then there is no issue at all. Only four or five are there. We can go inside in no time and can come back taking treatments. But the medications are expensive.” (I17SL)
‘Appropriateness and Ability to engage’: adherence and relationship between providers and patients	
Adherence to medications (Bangladesh)	“I stopped taking medicine before this for three or four years. I felt better at that time. After that I became sick again and I am taking.” IDI-BD-19 Age 40-49-M
Relationships with health care professionals	I: Do you have a good relationship with the doctors for example a healthy talk with each other? P: We can solve anything by taking them. But there is no time to chat like that. They also have a lot of patients to check. Therefore, they check quickly and prescribe the medications
Recommendations and unmet needs	
Recommendations (Sri Lanka)	I: There are patients like you who have hypertension. In your view, what can be done for them? To easier your lifestyle what are the things to be done? It can be from the hospital… P: Yes. If we can get treatments without a huge delay and if we can take all the drugs from the hospital if self… We don’t have money even to spend… Therefore, it’s good if we can have them. I: What else can be done? Even from the government what are the things to be done? P: Yes. Even if we get a small aid it’s very precious for us (Interview 1)

**Table 3:** Key themes with subthemes and key findings.

‘Approachability and Ability to perceive’: Patients’ awareness of hypertension and information sources	
Patients’ experiences of symptoms (if any) and beliefs	Overall, patients’ reported symptoms (or absence of them) across the three countries were quite similar, although perceptions of hypertension were mixed.
Patients’ awareness and knowledge of hypertension treatment, management and control	Most patients reported awareness of hypertension prior to diagnosis, but low knowledge of the specifics on how to prevent it or treat it. None of the interviewees reported being aware that hypertension is normally asymptomatic and there was little awareness of the possible consequences prior to being diagnosed.
Sources of Information drawn upon to inform decisions	Patients reported several sources of information available to them besides those provided by clinicians and community health workers including family members and friends, and the ‘hearsay”.
‘Acceptability and Ability to Seek’: the use of Ayurveda medicine and religious beliefs	
The Use of Ayurveda, alternative medicine, or informal treatment	Most participants who reported using Ayurveda, alternative medicine or homeopathy treatment revealed taking this medication together with the medication for hypertension.
Taking into account Religious beliefs when providing services	The lack of understanding and misinformation on how to handle hypertension during Ramadan was mentioned, particularly in Pakistan and Bangladesh with no participants reported being provided with advice on how to manage their condition during that period.
‘Availability and Ability to reach’: mounting difficulties in accessing services	
Long waiting times and shortages of medication.	The main barriers to accessing health services as reported by participants were the location of facilities, the absence of local services, poor quality facilities, long transportation, shortage of medications, and long waiting times. Patients also mentioned that cost was a barrier to accessing services and adhering to medication.
Lack of physical and human resources	Most patients in the three countries mentioned several difficulties in accessing services. These included: the location of the services and facilities, the absence of local services, long transportation and distance, the busyness of doctors, and the cost of the services.
‘Affordability and Ability to Pay’: out-of-pocket expenditure and merits of private vs public services	
Out-of-pocket expenditure	Most patients reported on the high cost of care, as well as the cost of medicines. The vast majority of respondents reported having no form of health insurance, which forced out-of-pocket payments for all services and medication.
Accessing services: Public versus Private	Most participants reported accessing the private sector for medication or small procedures with some refusing follow up in private facilities due to cost.
‘Appropriateness and Ability to engage’: adherence and relationship between providers and patients	
Adherence to medications	Most interviewees reported no problems in taking their hypertension medication. The majority of patients reported being prescribed medication and educated on life-style changes to mitigate hypertension, such as dietary modifications. However, there were also many reports of patients’ not adhering to the prescribed medication.
Relationships with health care professionals	Many patients reported on the importance of the provider-patient relationship and mentioned valuing doctors, who spent time with them, provided advice, and could be trusted. However, most patients reported that the experience with their doctor did not meet their expectations in government hospitals
Recommendations and unmet needs	
Recommendations (Sri Lanka)	Patients in the three countries reported welcoming health care outreach activities near their homes, desire for good quality local medical services, the need for access to doctors, medicine and diagnostics, and decreased of cost for medical services.

## Discussion

This qualitative study explored health system and population barriers to accessing hypertension care services in rural communities in Bangladesh, Pakistan and Sri Lanka from the patient’s perspective. A complementary objective of the study was to identify patients’ experiences of symptoms (if any) and management of hypertension, especially as it relates to adherence to pharmacologic therapy. Overall, most patients reported similar types of symptoms associated with diagnosis of hypertension across the three countries, indicating serious deficiencies in preventive hypertension screening when patients are asymptomatic. Patients’ perceptions of hypertension were mixed, as some patients downplayed the significance whilst others recognized it was a serious condition. For example, in Bangladesh, several participants described the consequences of hypertension in an alarming manner. Other qualitative studies [26] have suggested that patients with co-morbidities (i.e. diabetes) tended to focus on those, rather than on the symptoms and consequences of hypertension. Our findings suggest that while some patients reported serious concerns around being hypertensive, the majority, as described in the literature, had low awareness of its adverse consequences and on how to prevent or treat it.

Most patients in the three countries mentioned several difficulties in accessing services, many of them described with some level of frustration. The main barriers to accessing health services, as reported by participants, were dearth and poor quality of existing facilities, the busyness of doctors, long transportation, long waiting times, and medication shortages. Patients reported on the high cost of care and medicines, as well as on the economic opportunity and indirect treatment costs. Most participants relied on family members to help them overcome these barriers. When they could afford it, patients also mentioned overcoming barriers to medication shortages or long waiting times by accessing private providers or local pharmacies. Amongst patients who reported preferring private care to public, the majority mentioned quality of private healthcare as a motivating factor to access.

Patients in our study have reported many factors interacting and influencing adherence to hypertensive medication. These factors include the individual’s behavior and beliefs; the interaction of patients with the health system; the relationship between patients and health care professionals; and the effect of socioeconomic and cultural factors. Many interviewees in the three countries reported no problems in taking their antihypertensive medications. However, widely mentioned were experiences of lapses in adherence resulting from feeling better; only taking medication when they became symptomatic; beliefs that taking too much medicine for hypertension is not good; or simply forgetting to take medications on a daily basis, particularly when unplanned activities emerged. These results are in line with those reported in a systematic review exploring beliefs about consequences of taking hypertensive medication, where participants reported not needing treatment because they had no symptoms and also expressed fear of dependence to medications [7].

Medication cost as a barrier to adherence has been extensively reported in the literature [6, 7], with our study contributing to that knowledge. In our study, it is described in a more disquieting manner, since some respondents reported not taking any medication whatsoever due to financial constraints, especially in Bangladesh and Pakistan, where most classes of antihypertensive medications are not provided by the public sector. A few patients reported on facilitators for adherence, primarily the role of family members in supporting adherence to medications. Family members have been identified as playing a key role in improving adherence to medications in other studies from South Asia, and clearly are an effective resource [27].

Many patients reported on the importance of the provider-patient relationship and mentioned valuing doctors who spent time with them, provided advice, and could be trusted. However, most patients reported that, especially in government facilities, the experience with their doctor did not meet their expectations. Patients also reported on a lack of continuity of care and follow up with the providers who prescribed them medication. In our study, while there were few reports of doctors showing a familiar and caring attitude, the majority of patients complained that there was no time to form a relationship with the healthcare professional. These reports seem to suggest that there was no chance to develop trusting relationships as doctors were too busy. Their interactions were transactional rather than based on sharing information or behaving in a more familiar manner. Other studies have explored the importance of ‘bestowing trust’ on healthcare professionals and how these relationships develop. For example, a qualitative study exploring the experiences of hypertensive patients in Colombia identified a type of trust based on the Parsonian model, which is based on ‘deference’ and ‘asymmetry’ towards health care professionals [26]. Other studies, particularly in high-income countries, have identified other types of trust which are based on reciprocity (i.e. respect/empathy) and competence, which is believed to support adherence to treatment [28]. In our study, the only few reports of trusting relationships suggest both types were present, with more reports of trust based on reciprocity and empathy in the private sector, particularly among pharmacy shop owners.

An overarching theme that is present in many sections of the paper is participants’ unmet need for information. Patients reiterated on the importance of doctors spending time with them and voiced dissatisfaction with doctors who did not listen to their concerns. Patients also reported several misunderstandings due to not being properly informed. These included patients reporting they could be cured with no need for follow up or monitoring by a doctor, as well as beliefs around alternative medicine to control hypertension. In addition, many participants were not properly informed on lifestyle modifications and were not provided with advice on how to manage their condition during Ramadan or how to adapt their diet while fasting. Only few participants in the three countries could recall any prevention campaigns to increase knowledge on hypertension management. Overall, what transpires from these accounts is the lack of information and communication between patients and health care professionals. In the absence of this information, patients obtained knowledge through personal experience, from family members and friends, and ‘hearsay”.

Patients identified recommendations for better provision of hypertension prevention, treatment and management. Patients in the three countries reported a desire for good quality local medical services, the need for access to doctors, medicine and diagnostics, and decreased cost for medical services. Patients also described welcoming the prospect of greater health outreach activities into their communities, as these provide an opportunity to link rural and poor individuals to care, address the unmet need for greater prevention education, and lifestyle education and assessments. Participants highlighted their willingness to receive more training on hypertension management from CHWs in each country.

Patients also provided insight into suggestions outside of the health care system. Patients, mostly in Pakistan, often answered with a desire for increased access to clean water, electricity, and employment opportunities. Patients expressed frustration at many points with a public sector that is not properly functioning in rural areas and an unregulated private sector available to only a few. The private sector is part of the health systems in the three countries, and provides a myriad of mostly highly valued but prohibitively expensive services. Our findings underscore the need for new models of healthcare delivery in the public sector, such as the MCI with training physicians, as well as task shifting to non-physician healthcare providers for monitoring BP and health education, referrals, as well as additional support for those most disadvantaged with poorly controlled BP [8].

### Strength and limitations

To our knowledge, this is the first qualitative study exploring the health system and population barriers to accessing hypertension care services in rural areas in three low and middle-income South Asian countries from the patients’ perspectives. A strength of this paper is that semi-structured in-depth interviews have allowed us to explore patient’s challenges in accessing the health care system, as well as providing a diversity of experiences in seeking care for hypertension. This study has also attempted to operationalize Levesque et al’s framework [21], whilst allowing for new themes to emerge through inductive analysis. Adopting a broader and cumulative conceptualization of access to health care has facilitated a more in-depth analysis. Our analysis has highlighted the relevance of each dimension and more importantly how these adequately fit with the five ability dimensions related to patients. The strength of the conceptual framework consists of enabling the identification of factors that can have an impact on access and focuses on both, those related to the health system and those present at population level. It has also facilitated the emergence of issues that are normally not considered within the realms of access to services, but that have proven to be relevant. For example, by adopting the Levesque acceptation of access [21], we were able to identify how norms also permeate the health system and can relate to beliefs associated to modern versus traditional medicine or to culture and religion. By acknowledging that existent norms within the health system do not account for diverse practices, religions and cultures, one can act upon them and prevent increasing inequalities for some groups.

Two key limitations were identified in this study. First, we acknowledge that by presenting a comparative analysis of three countries, we lose some of the specificities of each country, as it is difficult to report all the relevant findings in one paper. Companion papers will be produced providing complementary experiential evidence at the country level on patient knowledge of hypertension; as well as the ways in which patients in each country seek, obtain and adhere to lifestyle modifications and hypertension medication through more detailed exploration of patients’ pathways. A second limitation of the study is that, although we attempted to include a breadth of participants from various groups, including those who are in marginal groups or isolated, our sampling strategy is likely to have under-selected those who are least connected to the health system. However, our sampling strategy of random selection of clusters with stratification by distance from government health facility and door-to-door sampling ensured wide representation of participants in all three countries, thereby enhancing the transferability of our findings.

## Conclusions

This qualitative study has identified barriers to accessing services in rural communities in Bangladesh, Pakistan and Sri Lanka from the patient’s perspective. Areas of improvement could focus on reorienting CHWs’ activities; involving family members in comprehensive counseling for medication adherence; providing appropriate training for health care staff to deliver effective information and services for controlling hypertension to patients; enhancing primary health care and specialist services; improving supplies of hypertensive medication in public facilities; taking into account patients’ cultural and social background when providing services; and facilitating access and treatment to those who are most vulnerable.

## Declarations

### Ethical approval and consent to participant

Ethical approval has been obtained from the Ethical Review Committees of the International Centre for Diarrhoeal Disease Research, Bangladesh (ICDDR, B) Aga Khan University, University of Kelaniya, London School of Hygiene & Tropical Medicine, (LSHTM), and Duke-NUS Singapore. All respondents were given an information sheet and were asked to sign and date a consent form. Consent was also obtained for audio-recording.

### Trial registration

The trial has been registered prospectively with clinicaltrials.gov (NCT02657746).

## Supporting information

S1 Table Interview topic guide. (DOCX)
