# Supplementary material for: Patients’ experiences on accessing health care services for management of hypertension in rural Bangladesh, Pakistan and Sri Lanka: A qualitative study
Source: PLoS One. 2019 Jan 25;14(1):e0211100. doi: 10.1371/journal.pone.0211100 (PMC6347162; doi:10.1371/journal.pone.0211100)
Supplement: S1 Table — (DOCX) [file pone.0211100.s001.docx]

Appendix 1 - Interview Topic Guide

| **Knowledge and Diagnosis** |
| --- |
| - Did you experience any symptoms before you were diagnosed with Hypertension? |
| - If yes, can you describe those symptoms of Hypertension? |
| - Do you have other health problems? |
| - Can you tell me about your other health problems (the one that worries you the most then follow up with others, then ask HTN and DM? |
| - How did you decide to seek care? Did the family help in the process? Or any organization? |
| - Can we talk about your experience of this process of seeking care? |
| - To what extent do you think HTN is an important disease? |
| - How much did you know about HTN before your diagnosis? What were your information sources at the time and now? |
| Prevention and Treatment |
| - Have you received any advice on preventive and control measures on HTN from Community Health Workers (CHWs)? |
| - Was the information received appropriate to your needs? |
| - Have you changed your behaviour (diet and exercise) since knowing about the condition? |
| - What was the treatment that was first prescribed? Was it subsequently changed? |
| - Does a family member or organization help you with taking the treatment? |
| - Did you have to pay anything out-of-pocket for the treatment or for travelling? |
| - Do you take other alternative medication? |
| - How were the health care facilities you visited? |
| - What difficulties you face during this process while seeking treatment? |
| - What in the process of treatment could have been handled better? |
| Access of Services and Receiving Care |
| - Are there shortages of drugs and consumables? Or access problems to facilities? Discuss problems. |
| - Are there problems accessing Home Health Education (HHE) and other services from CHWs? |
| - Are there problems accessing subsidies or financial support for additional health services? |
| Health Care Experiences and Recommendations |
| - How would you assess your communication with health providers you have encountered? |
| - Do you have a trusting relationship with your GP? |
| - To what extent have you been kept informed about your treatment? |
| - How can the Home Health Education and other services provided by CHWs be improved? |
| - Have you heard of any initiative to improve prevention of HTN? |
| - From your experience what could be done to make life easier for people suffering from HTN? |
| - Are there any changes that need to be made outside the health care system? |
